# Gingival Squamous Cell Carcinoma Predicted to Originate From the Gingival Sulcular Epithelium in a Young Female: A Report of a Rare Case

**DOI:** 10.7759/cureus.37046

**Published:** 2023-04-02

**Authors:** Shogo Kikuta, Yui Teratani, Katsuhisa Matsuo, Jingo Kusukawa

**Affiliations:** 1 Dental and Oral Medical Center, Kurume University School of Medicine, Kurume, JPN

**Keywords:** periodontal disease, young adult, mandibular gingiva, gingival sulcular epithelium, squamous cell carcinoma

## Abstract

Oral cancer is a disease primarily in older adults and extremely rare in young adults. Risk factors for oral cancer are irritants such as tobacco smoke and alcohol and chronic mechanical irritants but mechanisms involved in carcinogenesis in young adults are unclear because of less exposure to their risk factors. Herein, we report a rare case of gingival squamous cell carcinoma in a 19-year-old female patient, in whom the tumor predictably originated in the gingival sulcular epithelium. Histopathological examination of the resected tissue showed a cancer cell nest invading from the gingival sulcular epithelium without a breakdown of the basement membrane of the marginal gingival epithelium. Six years after the surgery, no recurrence or metastasis has been detected.

## Introduction

The prevalence of oral cancers is greater in older adults. Its risk factors include irritants such as tobacco smoke, alcohol, chronic mechanical irritation, poor oral hygiene practice, chronic ulceration from an ill-fitting denture, and others [1]. Although the number of young adults with oral cancer is minimal, it has increased yearly [2,3]. Given that exposure to oral cancer risk factors is less in young adults than in older adults, it is considered necessary to investigate the mechanisms or characteristics associated with carcinogenesis. Here, we report a rare case of a 19-year-old female patient with gingival squamous cell carcinoma, which may originate in the gingival sulcular epithelium.

## Case presentation

**Figure 1:**
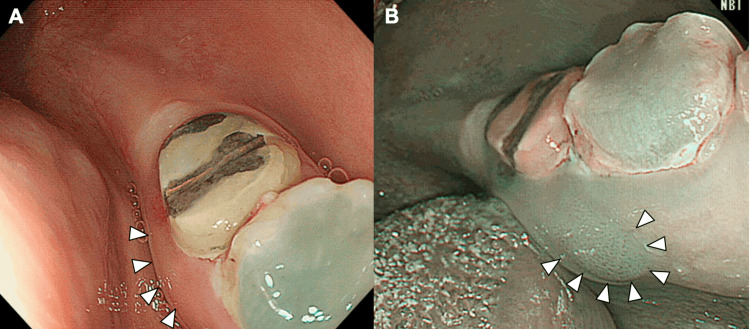
Intraoral tumor on the lingual aspect of the mandibular left second molar A. Intraoral tumor in the lingual gingiva. The tumor mass measured 10 × 5 mm (white arrowheads). B. NBI shows the accumulation of abnormal blood vessels in the tumor area (white arrowheads). NBI: narrowband imaging

A 19-year-old female patient was referred to our department with 5 x 10 mm gingival swelling on the lingual aspect of the mandibular left second molar. The gingival swelling was accompanied by contact pain, and papillary hyperplasia was observed in the gingival crevice on the lingual aspect (Figure 1A). The adjacent teeth had received the prosthetic procedure without subgingival margins and had no chronic irritation from the restoration. Narrowband imaging (NBI) showed abnormal blood vessels with intrapapillary capillary loops in the lingual gingiva (Figure 1B).

**Figure 2:**
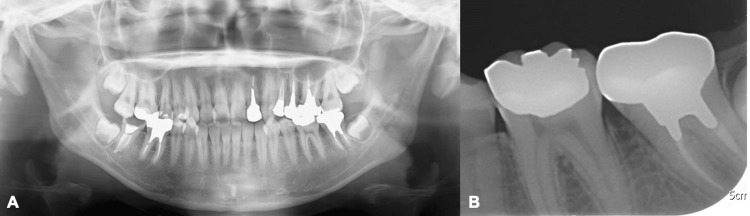
Preoperative X-ray images A. Preoperative panoramic imaging. B. Dental imaging. Alveolar bone resorption is found between the mandibular first and second molars.

Panoramic and dental radiographic images revealed alveolar bone resorption between the mandibular left first and second molars (Figures 2A, 2B).

**Figure 3:**
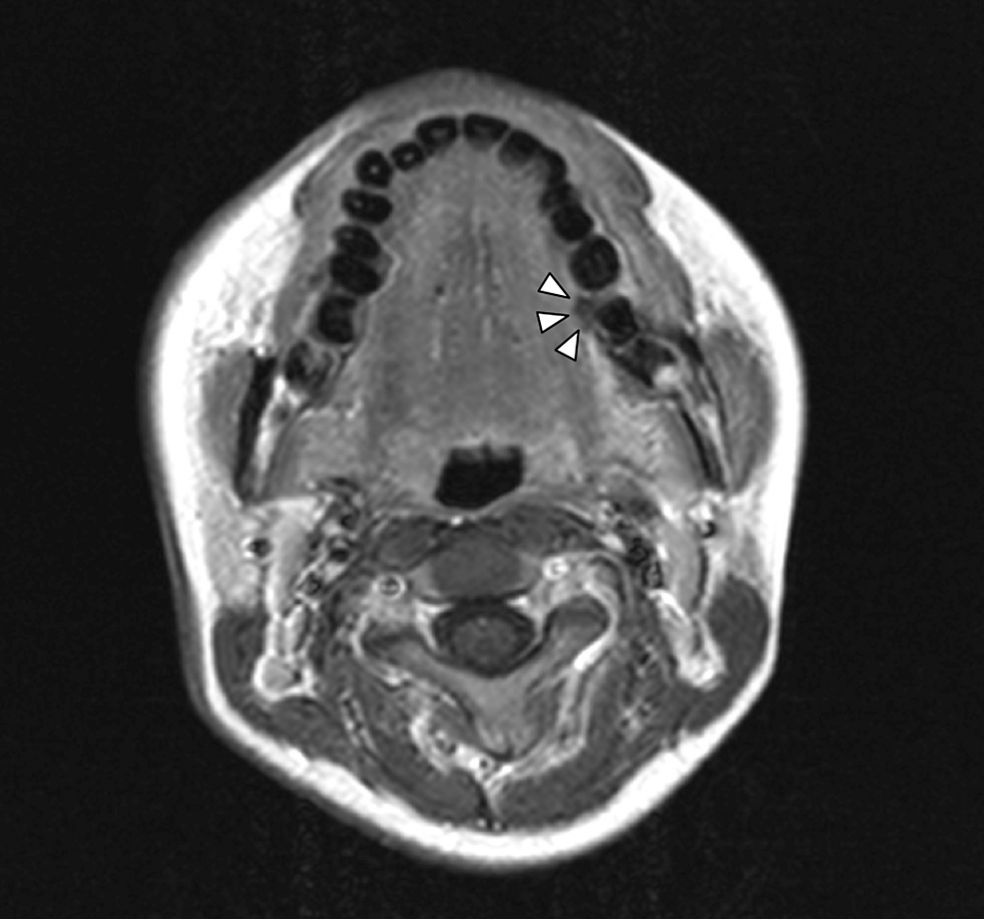
MRI image Contrast-enhanced T1-weighted MRI shows a mass with a slight contrast effect in the lesion area (white arrowheads).

The presence of a dental prosthesis prevented contrast-enhanced CT scans from visualizing the tumor. Magnetic resonance imaging (MRI) captured a mass with a slight contrast enhancement effect at the lesion (Figure 3).

**Figure 4:**
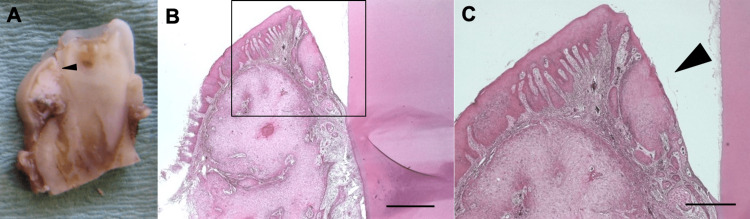
Histopathological specimens A. Tumor resected and fixed in formalin. Tumor formation was observed in the gingival sulcular epithelium (black arrowhead). B, C. Histopathological specimens showed follicular cell differentiation extending from the gingival crevicular epithelium to the subbasement membrane infiltration. Cancerous growth from the inner margin of the gingival sulcus is observed. The epithelium of the outer margin was preserved (black arrowhead). B) 10 × magnification. The scale bar indicates 1 mm. C) 20 × magnification. The scale bar indicates 500 μm.

No signs of metastasis to lymph nodes or any other tissues or organs were detected. The result of the tissue biopsy showed a highly differentiated squamous cell carcinoma. The patient was diagnosed with squamous cell carcinoma (SCC) of the left mandibular gingiva (cT1N0M0). The patient was treated by excisional surgery to remove the entire tumor along with a safety margin of surrounding tissues above the mandibular canal, involving the mandibular bone, gingiva, and tooth. After marginal resection of the mandible, the wound had primary closure. Histopathological examination of the tissue resected during the procedure did not show any breakdown of the basement membrane of marginal gingival epithelium. However, it revealed an invasion of a cancer cell nest under the basement membrane extending into the gingival sulcular epithelium (Figures 4A-4C).

**Figure 5:**
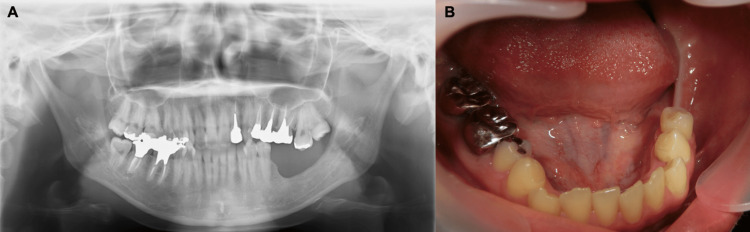
Postoperative findings A. Panoramic image taken six years after surgery B. Intraoral photographs taken six years after surgery

The pathological diagnosis was mandibular gingival SCC (T1N0M0). As the resected specimen showed histologically negative margins, follow-up was performed without adjuvant therapy. After six years of the surgery, no sign of recurrence or metastasis is observed (Figures 5A, 5B).

## Discussion

The rate of oral cancer is the highest among persons aged 60-80 years and slightly higher in males than females [4]. Although the incidence of cancers in the oral cavity is rare among persons under the 40s, its incidence in the younger generation has been increasing in recent years [2]. The incidence of cancers in the oral cavity in young patients per year has increased more than five times since 1950 [3]. Carcinogenesis studies in the younger population report that female patients tend to have no apparent etiologic factors compared to male patients [5-8]. Alcohol and smoking have been considered to be typical risk factors for oral cancers. However, in contrast, though smoking and alcohol use in the young population has been declining over the years, the incidence of oral cancer in them has been increasing [9]. Thus, it has been reported that the association between these factors and carcinogenesis may not be as strong as thought [10]. Genetic predisposition is also possible, especially in patients with no recognized risk factors such as young adults [11]. Compared to older patients, a significant increase in chromosome fragility has been revealed after exposure to mutagenic agents [11]. This fragility could lead to genetic abnormalities [11]. In addition, there is a clear and significant relative risk of SCC in first-degree relatives of head and neck cancer patients [12]. Another possible factor in oral cancer in young people is that chronic immunodeficiency states may play an essential role in carcinogenesis in young people [13]. The patient's family history is unknown, but there are no signs of immunodeficiency in the blood-draw data available.

Periodontal disease, a chronic inflammatory disease, may be linked with developing cancers in the oral cavity [14,15]. In particular, the potential involvement of periodontopathogenic bacteria has been suggested, with Porphyromonas gingivalis and Fusobacterium nucleatum as the possible main pathogenic bacteria involved in developing oral cancer [16-18]. It has been shown that oral cancer progression was promoted in mouse models chronically infected with these bacteria [19]. In the present case, however, there was no prominent finding of periodontal disease, which indicates the absence of an association between the pathogenic bacteria and the development of oral cancer, at least for this patient.

The tongue accounts for most oral cancer sites, followed by the gingiva [4]. The location of cancer initiation in this patient may be pathologically the sulcular epithelium, as the basement membrane of the outer gingival epithelium was preserved, and the cancer cell nests extending into the gingival sulcular epithelium were observed. We searched PubMed and Google Scholar for case reports of gingival squamous cell carcinoma originating from the gingival sulcular epithelium published before March 2023. The search terms included “oral cancer,” ”carcinoma,” “squamous cell carcinoma,” and “gingival sulcular epithelium.” There had been only one report of oral cancer originating in the oral sulcular epithelium [20]. The reason might be that cancer cells in this area spread beyond the gingival sulcus as cancer grows, making it difficult to determine where it originated. As a result, it is often difficult to pinpoint the exact site of carcinogenesis for gingival cancer. Further research and data accumulation may be necessary to elucidate the development of oral cancers in young populations without known risk factors.

## Conclusions

This case report presented a rare case of squamous cell carcinoma originating from the gingival sulcular epithelium in a 19-year-old female. The patient in this report developed SCC despite no history of exposure to risk factors such as smoking, drinking, and periodontal disease. Furthermore, this patient is a rare case in which we may determine pathologically that it originated in the gingival sulcular epithelium. Further research is needed to learn more about the exact site and cause of carcinogenesis.
